# Effect of a single dose of letrozole on ejaculation time, semen quality, and testicular hemodynamics in goat bucks subjected to heat stress

**DOI:** 10.1007/s11259-024-10551-5

**Published:** 2024-10-09

**Authors:** Ola Adel, Hossam R. El-Sherbiny, Abdallah M. Shahat, Sayed Taha Ismail

**Affiliations:** Theriogenology Department, Faculty of Veterinary Medicine, Cairo University, 12211 Giza, Egypt

**Keywords:** Climate change, Aromatase inhibitor, Doppler ultrasonography, Reproductive hormones, Male fertility

## Abstract

Letrozole (LTZ) is an aromatase inhibitor that limits estrogen (E2) production and increases testosterone (T) levels. This research aimed to examine the impact of a single dose of LTZ on testicular hemodynamics, ejaculation time, and semen quality in goats under heat stress (HS). Therefore, Doppler examination and semen evaluation were performed on twelve mature bucks for two weeks (W-1, W-2) as pre-heat stress control during winter. Then during summer HS bucks were subjected to Doppler examination, semen evaluation, and hormonal analysis (T, E2, and LH) at 0 h. Afterward, bucks were assigned into two groups and subcutaneously injected with physiological saline (*n* = 6; CON) or LTZ (0.25 mg/kg BW; *n* = 6; LTZ). Both groups were subjected to Doppler scanning and hormonal analysis at 2, 4, 24, 48, 72, 96,144, and 168 h. Semen evaluation was performed at 48 and 168 h. The LTZ group showed increasing (*P* < 0.05) in semen volume, sperm motility, and viability and decreasing (*P* < 0.05) in ejaculation time and sperm abnormalities compared to CON group at 48 h. Additionally, T concentrations increased (*P* < 0.01) at 2, 24, and 48 h, E2 decreased (*P* < 0.01) from 2 to 48 h, and LH raised (*P* < 0.01) at 2 and 72 h in LTZ group compared to CON one. Doppler indices reduced (*P* < 0.05) at 96 h in LTZ group. semen pH and scrotal circumference were not affected by LTZ. In conclusion, LTZ administration shortened ejaculation time and enhanced semen quality in bucks during HS.

## Introduction

One of the most difficult aspects of climate change is the rise in global temperature, which is predicted to rise by 4 °C during the next 100 years (Nanas et al. 2020). Climate change impairs animal productivity, due to increasing competition for natural resources, shortage of feed quality and quantity, expansion of livestock diseases, and loss of biodiversity (Rojas-Downing et al. 2017).

It’s well known that testosterone (T) and estrogen (E2) participate in testicular function and modify oxidative stress by testicular antioxidant enzymes. T regulates several physiological processes in the testis such as blood-testis barrier conservation, connecting between Sertoli cell and spermatid, in addition to the antiapoptotic role and releasing of sperm cells (Kim et al. 2017; Roque et al. 2020). E2 plays a role in germ cell survival and Sertoli cell proliferation (Rosati et al. 2019; Yan et al. 2020). Heat stress (HS) causes detrimental effects on testicular activity leading to a reduction in T production (Li et al. 2016; Hasani et al. 2020). On the contrary, increasing E2 levels exhibit negative feedback inhibition of the hypothalamic-pituitary axis, ultimately lowering follicular stimulating hormone (FSH) and luteinizing hormone (LH) (Hou et al. 2020). Therefore, the imbalance between E2 and T concentrations leads to the malfunctioning of Leydig cells and spermatogenic defects(Adamczewska et al. 2022). Furthermore, disturbs the epididymal sperm and testicular germ cells (Li et al. 2024), reducing sperm motility and production as well as increasing the number of morphologically aberrant spermatozoa consequently lowering sperm quality (Nowicka-Bauer and Nixon 2020). Additionally, high temperature causes tissue hypoxia due to the insufficient blood supply to the testes, ensuing inability to compensate for the increase in tissue metabolism (Paul et al. 2009; Hamilton et al. 2016). Subsequently, triggering detrimental effects on the testes and sperm quality (Aitken et al. 2016; Kastelic et al. 2017; Houston et al. 2018; Leite et al. 2022).

Following plenty of studies, HS assessment primarily depends on temperature humidity index (THI) calculation (Habeeb et al. 2018). Ruminants that kept in THI over 75 are considered under HS circumstances (Hahn et al. 2009). Alleviation of negative impacts of HS is beyond crucial for optimum reproductive competence. Huge efforts have been spent to improve male reproductive performance under HS by using antioxidants in goats (El-Sherbiny et al. 2022a), rams( El-Sherbiny et al. 2022b; Shalofy et al. 2023) or by using hormones such as GnRH agonist (Giriboni et al. 2019).

Aromatase is a cytochrome P450 enzyme that is involved in the conversion of androstenedione and T into E2, thus aromatase inhibitors interfere with the aromatase in E2-secreting tissues to reduce the E2 production and increase T levels either directly by accumulating T that not changed to E2 or indirectly by permitting gonadotropin secretion via the negative feedback on the hypothalamic-pituitary axis, as a result improving male fertility (Dias et al. 2016). Additionally, aromatase inhibitors were effective in the treatment of endometriosis, breast cancer, and spermatogenesis disorders (Garzon et al. 2020; Bradley et al. 2022; Yang et al. 2022). Letrozole (LTZ) is a non-steroidal third-generation aromatase inhibitor that binds with the heme iron of cytochrome to hinder aromatase activity (Mukherjee et al. 2022). Previous studies have shown that LTZ improved semen quality in Markhoz goats (Rezaei et al. 2022), roosters (Ali et al. 2017), rats (Hmood Jassim et al. 2022), and infertile men (Peivandi et al. 2019; Jahan et al. 2021; Kooshesh et al. 2022; Akinyele et al. 2022).

To date, studies on LTZ administration in stressful conditions such as HS are limited. Since HS deteriorates testicular function and reduces T concentrations, considering the major role of T in semen quality; the authors hypothesized that LTZ would improve ejaculation time, semen parameters, and testicular blood flow in bucks during HS conditions. Therefore, the present study aimed to investigate the acute effect of LTZ administration (single dose, S/C) on the reproductive functions of Baladi bucks exposed to summer HS via assessment of the testicular hemodynamics, ejaculation time, and semen quality.

## Materials and methods

### Ethical approval statement

This work was accepted by the animal ethics committee at the Faculty of Veterinary Medicine, Cairo University (Protocol code: Vet CU 09092023747).

### Animals’ management and experimental design

Twelve Baladi goat bucks, aged between 2.5 and 3.5 years, with an average body weight of 38.5 ± 1.4 kg, were used in the current study. Animals were housed at the research farm of the Theriogenology Department, Faculty of Veterinary Medicine, Cairo University, Egypt. Before including in the experimental procedures, bucks were checked to exclude any buck suffering from any cardiovascular or andrological problems through general inspection of the vital signs and Doppler ultrasound examination of the reproductive system. They were kept in a paddock with the availability of a 20 m^2^ shaded slatted area and exposed to natural daylight, ambient temperature, and relative humidity during winter and summer seasons in Egypt. They also maintained a balanced diet following the NRC instructions (2007), composed of 400 g/ head/ day of ready-to-eat pelleted concentrates and 1.25 kg/head/day of green roughage. Primarily, Doppler examination and semen evaluation were performed on twelve bucks for two weeks as pre-heat stress control (W-1 and W-2) during winter (February 2023). Then, during summer HS conditions (June 2023) all bucks were subjected to Doppler examination, semen evaluation, and blood sampling for hormonal analysis (T, E2, and LH) at 0 h. Afterward, they randomly divided into two groups as follows: (1) the control group (*n* = 6; CON) received 0.3% hydroxypropyl cellulose in 0.9% NaCl solution without letrozole (2) letrozole group (*n* = 6; LTZ) received a single dose of LTZ (S/C; 0.25 mg/kg BW) (Sigma-Aldrich, USA; CAS- No:112809-51-5). The LTZ treatment was prepared using 0.3% hydroxypropyl cellulose (Sigma-Aldrich, USA) in 0.9% NaCl solution with adding LTZ to obtain the required concentration, the final injection volume for each buck was two ml (Rezaei et al. 2022). Then both groups were subjected to Doppler scanning and blood sampling for hormonal analysis (T, E2, and LH) at 2, 4, 24, 48, 72, 96,144, and 168 h. While, semen collection and evaluation were performed at 48 and 168 h.

### Assessment of HS and physiological response

**Fig. 1:**
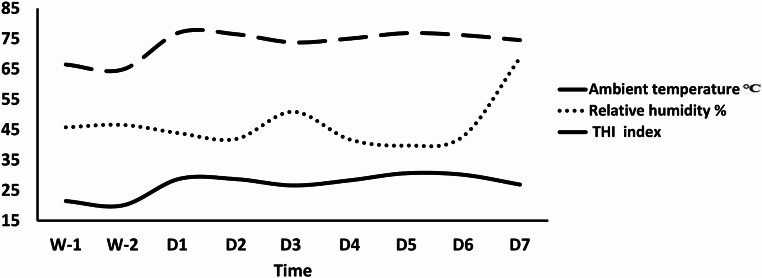
Temporal changes of the ambient temperature, relative humidity, and temperature humidity index (THI) throughout the study timeline in Giza city, Giza governorate, Egypt. The Calculated THI was based on the equation proposed by Kendall and Webster (2009) THI= (1.8 × T + 32) − [(0.55 − 0.0055×RH) ×(1.8×T − 26)]. W-1 and W-2 referred to pre-heat stress conditions and from (Day; D1 to D7) referred to hot humid conditions

Temperature and relative humidity were calculated daily from the Egyptian meteorological authority and territory (Giza city, Giza governorate, Egypt) based on the ambient temperature and relative humidity during the pre-heat stress period (20-21.4 ^o^C and 45.8–46.6%) and during HS period (26.6–30.7 ^o^C and 40-68.8%). According to Kendall and Webster (2009), THI = (1.8× T + 32)- [(0.55-0.0055×RH) × (1.8×Temperature-26)]; therefore, if THI < 70 indicates minimum HS, 70–80 indicates moderate HS, and > 80 indicates severe HS (El-Tarabany et al. 2017). Herein, THI values during the pre-heat stress period (W-1 and W-2) ranged from 64.9 to 66.5; thus, bucks were under minimum HS, while during experimental time points of hot humid conditions ranged from 74 to 77.1 (Fig. 1). Consequently, the studied bucks suffered from moderate HS conditions (El-Tarabany et al. 2017).

Physiological parameters such as rectal temperature, respiratory rate, and heart rate were recorded at each examination. Rectal temperature was noted by placing a clinical thermometer into the rectum for one minute. The respiratory rate was achieved by counting the flank motions for one minute. Additionally, heart rate was measured by applying the stethoscope near the left axillary bone, and then recording the heart rate for one minute.

### Blood sampling and hormonal analysis

After overnight fastening and at the early morning (9 a.m.) just before each ultrasonographic examination, blood samples were collected from the jugular vein and placed into plain tubes. All samples were centrifuged for 15 min at 3000 rpm. Serum samples were harvested and stored at -20 ℃ for further assessment of T (ng/mL), E2 (pg/mL), and LH (mIU/mL). T was measured by commercial ELIZA (DiaSino Laboratories Co., China, respectively) kits using a microplate ELIZA reader (SCILOGEX^®^, Rocky Hill, CT, USA) referring to the manufacturer’s instructions. The optical density (OD) was assessed at 450 nm (using 620 to 630 nm as the reference wavelength to reduce well imperfection). The intra- and inter-assay coefficients of variation were 3.3 and 4.8%, respectively and the assay sensitivity was 0.055 ng/ml. E2 and LH concentrations were measured by (YHLO iFlash-E2 immune assay analyzer, Netherlands and Siemens Advia centaur system, Tarry town, USA, respectively) with precision of ≤ 10% and ≤ 20 total coefficient variation.

### Evaluation of testicular blood flow

**Fig. 2:**
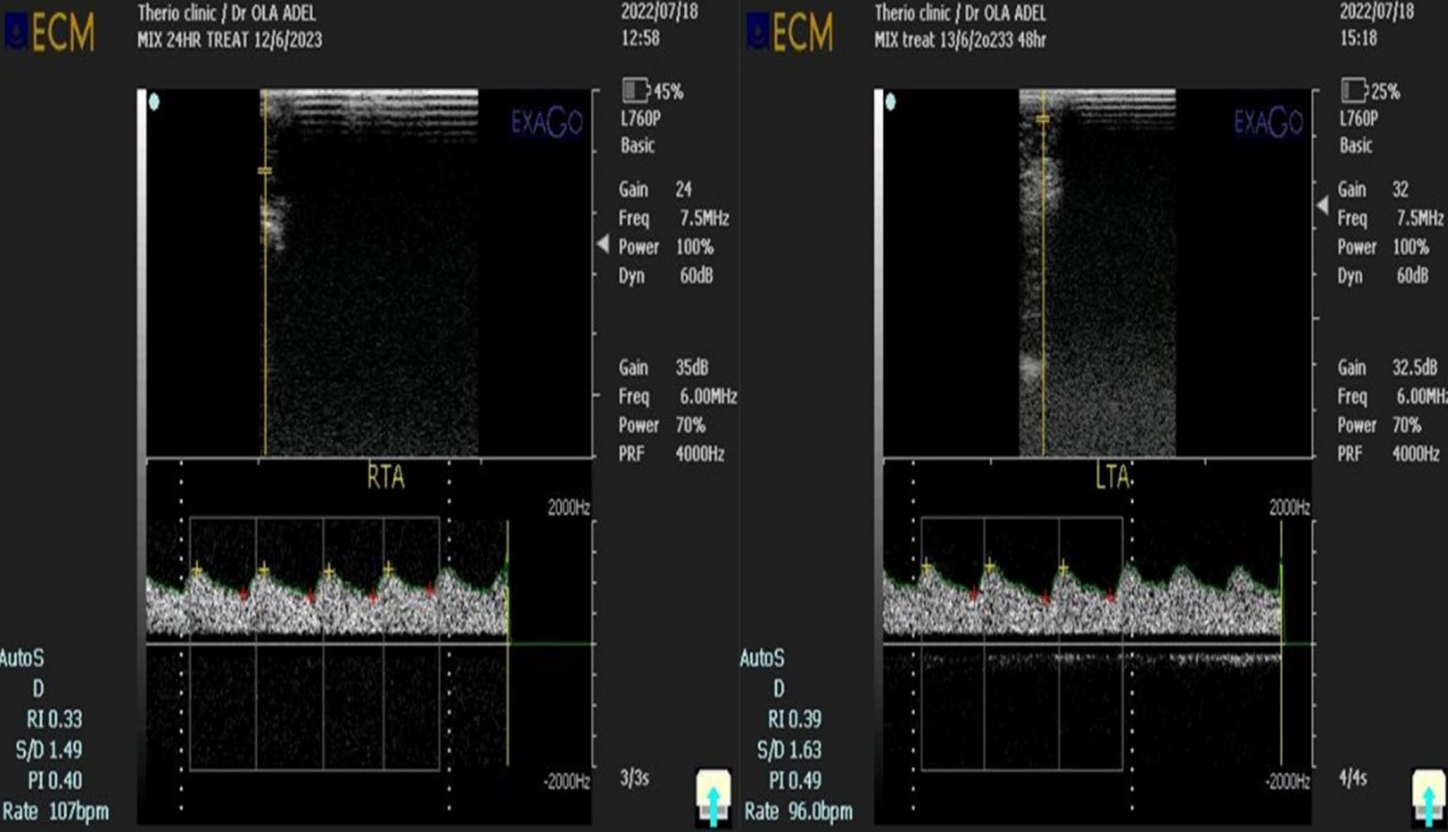
The spectral waveform of supratesticular artery by pulsed Doppler ultrasonography to assess parameters as resistive index, pulsatile index, and systolic/diastolic ratio (RI, PI, and S/D, respectively)

Before Doppler examination, all animals were secured without sedation to eliminate their impact on testicular hemodynamics. Also, the scrotal hair on the testes and spermatic cord was removed to reduce the artifacts. Gel was applied to the transducer before each examination. By using Doppler ultrasonography (EXAGO, Echo Control Medical, France) equipped with a 5–7.5 MHz linear array probe to evaluate the testicular hemodynamics. Doppler examinations were done daily at 9.00 a.m. by the same operator. For pulsed wave measurements, Doppler sets as the gate (0.5 mm), filter (50 Hz), and the angle between the Doppler beam and the longitudinal axis of supratesticular artery (STA; < 60°) were accustomed and maintained constant through the study. Doppler probe was positioned vertically on a side of each scrotum and displaced upward till reaching the STA at the proximal pole of the testis within the vascular network (El-Sherbiny et al. 2022c). Following the appearance of the spectral waveform of STA, parameters were assessed as resistive, systolic/diastolic ratio, and pulsatility index (RI, S/D, and PI, respectively; Fig. 2).

### Measuring scrotal circumference

Scrotal circumference (SC) was measured through the experimental period by using a tape measure graduated in cm.

### Semen collection and evaluation

Semen was collected by an electeroejaculator (ELECTROJAC^TM^III 6–15 V, IMV, France) at 0, 48, and 168 h and directly sent to the laboratory for evaluation. At each semen collection, ejaculation time (the elapsed time between the beginning of the electro-stimulation and ejaculation) was recorded using a stopwatch (Ogundele et al. 2016). Ejaculates were transferred into pre-warmed graduated tubes kept in water bath 35 ℃ and its volume was measured to the nearest 0.1 mL. A digital pH meter (JENWAY, model:3510, UK) was used to assess the seminal pH. Mass sperm motility was performed by placing a dense drop of undiluted semen sample on a pre-warmed (37 ℃) glass slide without a coverslip and examined using a light microscope (10 x magnification) (Labo America, Inc. U.S.A.). The score was given from 0 (no motion) up to 5 (highly forceful motion) depending on the powerfulness of the wave motion of the collected spermatozoa. For sperm individual motility, a diluted semen sample [sodium citrate dihydrate 2.9% (1:20; v/v)] was placed on a pre-warmed glass slide (37 °C) and cover-slipped; the percentage was estimated for the spermatozoa that revealed forward progressive motility. Additionally, eosin-nigrosine-stained semen smears were performed to estimate the percentage of sperm viability and sperm cell morphology (Dott 1956) by using a light microscope under an oil immersion lens. For sperm viability (200 sperm cells were assessed/slide), the alive spermatozoa excluded the eosin stain and appeared colorless, while dead spermatozoa absorbed the eosin stain and appeared pinkish. Also, the abnormalities in the head, neck, mid-piece, and tail were recorded for normal morphology assessment (200 sperm cells were assessed/ slide). Duplicate smears were checked for all the studied semen parameters. The same expert technician performed all evaluations throughout the experimental period to verify consistency.

### Statistical analysis

Shapiro–Wilk test was used to confirm whether all data was normally distributed or not. As there were no significant differences in Doppler ultrasonographic readings between the right and left testes, the data for each buck was pooled. The treatment effect (LTZ versus CON), time 0, 2, 4, 24, 48, 72, 96, 144, and 168 h, and the treatment*time interaction on testicular Doppler parameters (RI, S/D, and PI), hormonal analysis, ejaculation time, and semen parameters were analyzed using a repeated measure two-way ANOVA followed by Bonferroni post hoc test. T-test was applied to compare between the two groups (CON and LTZ) at each sampling points throughout the studied parameters. Statistical Package for Social Sciences SPSS^®^ (SPSS Inc., Version 25.0, Chicago, IL, USA) was used for data analysis, moreover, probability equal to or less than 5% was considered a significant result. The values were presented as means ± standard error of means (SEMs).

## Results

### Effect of LTZ on the physiological parameters

Heart rate revealed time (*P* < 0.01) and time*treatment interaction (*P* < 0.05) post LTZ administration. Heart rate was reduced (*P* < 0.05) at 2 h and 96 h compared to the CON group. there was time * treatment interaction (*P* < 0.05) at rectal temperature. In LTZ group, rectal temperature increased (*P* < 0.05) at 4 h and 72 h compared to CON one. However, respiratory rate showed no significant difference among both groups (Table 1).

**Table 1:** Effect of letrozole on physiological traits in Baladi goats during pre-heat stress and heat stress conditions. Data was obtained as mean ± SEM

Time	Heartrate (beats/min) CON LTZ	*P*-value	Respiratory rate (breaths/min) CON LTZ	*P*-value	Rectal temperature (℃) CON LTZ	*P*-value
W-1	117.96 ± 13.53 117.71 ± 13.65	0.99	23.83 ± 2.77 23.00 ± 2.77	0.80	39.3 5 ± 0.18 39.35 ± 0 0.15	0.22
W-2	91.76 ± 7.65 91.68 ± 7.70	0.99	25.00 ± 2.70 23.83 ± 2.77	0.77	39.45 ± 0.18 39.15 ± 0.17	0.26
0 h	109.34 ± 6.92 102.00 ± 7.35	0.48	25.33 ± 3.60 25.33 ± 4.76	1.00	39.67 ± 0.07 39.63 ± 0.05	0.73
2 h	118.25 ± 10.40 90.85 ± 1.63*	0.04	30.67 ± 5.49 39.67 ± 6.98	0.51	39.36 ± 0.11 39.67 ± 0.07	0.05
4 h	118.25 ± 10.40 97.76 ± 2.25	0.08	30.67 ± 5.49 32.67 ± 5.52	0.80	39.36 ± 0.11 39.70 ± 0.05*	0.03
24 h	101.55 ± 6.10 87.87 ± 5.59	0.13	25.33 ± 3.47 30.00 ± 5.70	0.50	39.53 ± 0.12 39.46 ± 0.10	0.60
48 h	99.18 ± 7.17 85.74 ± 5.22	0.16	21.50 ± 2.04 29.33 ± 5.82	0.25	39.65 ± 0.16 39.46 ± 0.12	0.39
72 h	91.56 ± 7.37 89.91 ± 3.39	0.84	21.33 ± 2.01 25.33 ± 1.68	0.15	39.26 ± 0.07 39.63 ± 0.10*	0.01
96 h	78.11 ± 2.84 101.73 ± 6.00*	0.005	33.33 ± 4.86 28.67 ± 2.95	0.43	39.36 ± 0.22 39.67 ± 0.23	0.37
144 h	78.11 ± 2.84* 90.97 ± 3.57	0.01	33.33 ± 4.86 36.33 ± 7.96	0.75	39.36 ± 0.22 39.67 ± 0.05	0.22
168 h	109.34 ± 6.92 104.67 ± 7.36	0.65	25.33 ± 3.60 31.33 ± 7.36	0.55	39.67 ± 0.07 39.46 ± 0.11	0.16

*LTZ* letrozole group, *CON* control group, *h* hours. *Refers to a significant difference (*P* < 0.05) between groups (LTZ vs. CON) at that time. W-1 and W-2 denoted to pre-heat stress examination and from 0 h to 168 h denoted during heat stress

### Effect of LTZ on the level of T, E2, and LH concentrations

**Fig. 3:**
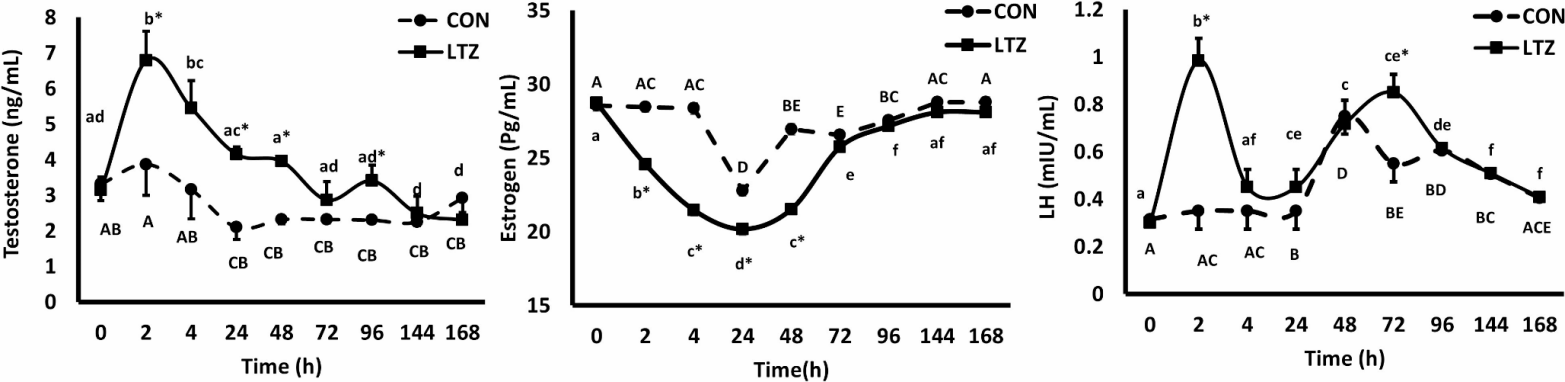
Alterations in serum levels of testosterone (ng/mL), estrogen (Pg/mL), and luteinizing hormones (LH; mIU/mL) in Baladi bucks that received letrozole (*n* = 6; LTZ) compared to the control (*n* = 6; CON) group during summer heat stress. Values are presented as means ± SEM. ^A−E^ in the control group and ^a−f^ in the letrozole group without a common superscript denotes a significant difference (*P* < 0.05) within that group. * means a significant difference (*P* < 0.05) between the two groups (LTZ vs. CON ) at the same time point. There was a time effect (0.001), treatment (0.001), and time*treatment interaction (0.001) for all measured hormones

The effect of LTZ administration on the levels of T, E2, and LH at the experimental time points (0, 2, 4, 24, 48, 72, 96, 144, and 168 h) in Baladi bucks is presented in Fig. 3. There was a time (*P* < 0.01), treatment (*P* < 0.01), and treatment*time interaction (*P* < 0.01) effect in the serum levels of all measured hormones. In the LTZ group, T concentrations showed a marked increase (*P* ˂ 0.05) in LTZ group at 2 h (6.79 ± 0.82), 24 h (4.15 ± 0.20), and 48 h (3.95 ± 0.07) post-injection compared to CON group. Concurrently, E2 decreased (*P* < 0.01) from 2 h (24.56 ± 0.33) till 48 h (21.51 ± 0.33). Nevertheless, LH elevated (*P* < 0.01) at 2 h and 72 h (0.98 ± 0.09 and 0.85 ± 0.07) in LTZ group compared to CON one.

### Effect of LTZ on testicular hemodynamics, SC, and ejaculation time

**Fig. 4:**
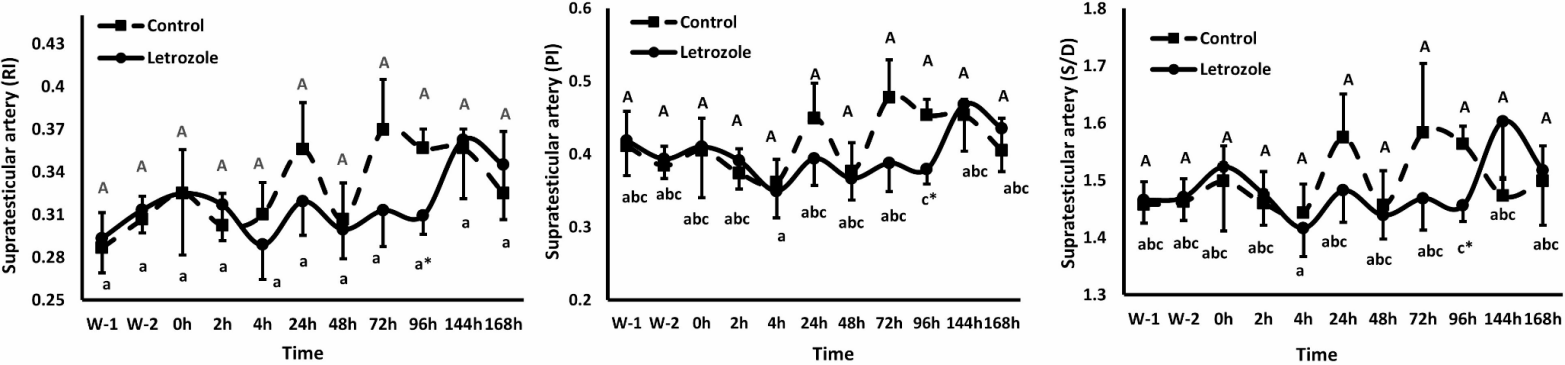
Changes (Mean ± SEM) in the Doppler indices: resistive index (RI), pulsatility index (PI), and systolic/diastolic ratio (S/D) in Baladi bucks given 0.25 mg/kg Letrozole S/C (*n* = 6) and control bucks given 2 mL physiological saline (*n* = 6) during heat stress. Values are presented as means ± SEM. ^A^ in the control group and ^a−c^ in the letrozole group without a common superscript denotes a significant difference (*P* < 0.05) within that group. * means a significant difference (*P* < 0.05) between the two groups (LTZ vs. CON) at the same time point. W-1 and W-2 denoted the pre-heat stress period and from 0 h to 168 h denoted summer heat stress

The effect of LTZ administration on testicular blood flow throughout the experimental time points is shown in (Fig. 4). Overall, there was no effect of time, treatment, and time*treatment interaction (*P* > 0.05) for Doppler indices (RI, S/D, and PI). However, RI, S/D, and PI were reduced (*P* < 0.05) in LTZ group compared to CON one at 96 h. In addition, SC was not influenced either by LTZ administration, time, or their interaction (*P* > 0.05) as presented in Table 2. Furthermore, ejaculation time was affected by LTZ administration (*P* < 0.01), time (*P* < 0.01), and LTZ administration*time interaction (*P* < 0.01). Ejaculation time reduced (*P* < 0.01) in LTZ group compared to the CON one throughout the experimental time points (48 and 168 h) post-LTZ administration (Table 2).

**Table 2:** Scrotal circumference and semen characteristics in the treated group (LTZ; 0.25 mg/kg BW; *n* = 6) compared to the control bucks (CON; 2 mL physiological saline; *n* = 6). Data was obtained as mean ± SEM

Parameters	Pre-heat stress period Average of two times (W-1 and W-2)	Heat stress period 0 h 48 h 168 h			*P*- value
Scrotal circumference (cm) CON LTZ	24.40 ± 0.20^A^ 24.70 ± 0.10^a^	24.40 ± 0.20^A^ 24.70 ± 0.10^a^	24.40 ± 0.20^A^ 24.70 ± 0.10^a^	24.40 ± 0.20^A^ 24.70 ± 0.10^a^	1.00
*P*-value	0.31	0.31	0.31	0.31	-
Ejaculation time (Seconds) CON LTZ	23.41 ± 0.74^A^ 23.41 ± 0.58^a^	35.80 ± 2.53^B^ 38.30 ± 2.51^b^	35.30 ± 2.60^B^ 24.30 ± 1.50^a*^	36.00 ± 2.50^B^ 25.80 ± 0.60^a*^	0.001
*P*-value	1.00	0.50	0.005	0.009	-
Volume (mL) CON LTZ	1.65 ± 0.02^A^ 1.55 ± 0.05^a^	0.87 ± 0.22^B^ 0.80 ± 0.20^b^	0.50 ± 0.08^B^ 1.50 ± 0.05^a*^	0.60 ± 0.10^B^ 1.00 ± 0.10^b^	0.001
*P*-value	0.14	1.00	0.001	0.10	-
pH CON LTZ	6.90 ± 0.03^A^ 6.88 ± 0.04^a^	6.90 ± 0.04^A^ 7.00 ± 0.02^b^	6.90 ± 0.02^A^ 6.90 ± 0.02^b^	7.00 ± 0.02^A^ 7.06 ± 0.03^b^	0.39
*P*-value	0.42	0.16	0.77	0.30	-
Mass motility (Score 0–5) CON LTZ	3.83 ± 0.24^A^ 3.91 ± 0.12^a^	3.58 ± 0.30^AC^ 3.91 ± 0.23^a^	3.00 ± 0.20^BC^ 4.20 ± 0.10^a*^	3.58 ± 0.30^AC*^ 2.67 ± 0.21^b^	0.01
*P*-value	0.77	0.40	0.002	0.03	-
Individual motility (%) CON LTZ	84.16 ± 2.86^A^ 84.16 ± 2.38^a^	73.33 ± 3.30^B^ 72.50 ± 3.80^b^	67.50 ± 3.80^B^ 83.33 ± 1.00^ac*^	67.50 ± 3.80^B^ 59.10 ± 3.70^d^	0.001
*P*-value	1.00	0.87	0.008	0.15	-
Viability (%) CON LTZ	91.41 ± 0.77^A^ 91.00 ± 0.74^a^	81.50 ± 2.40^B^ 81.60 ± 2.90^bc^	78.50 ± 2.30^B^ 84.33 ± 0.80^b*^	79.83 ± 2.65^B^ 77.50 ± 2.64^c^	0.001
*P*-value	0.70	0.96	0.03	0.54	-
Normal Morphology (%) CON LTZ	90.16 ± 0.96^A^ 88.83 ± 0.93^a^	81.83 ± 1.53^B^ 82.83 ± 1.37^b^	85.50 ± 0.67^C^ 88.50 ± 0.42^a*^	84.83 ± 0.87^BC^ 86.50 ± 1.33^a^	0.001
*P*-value	0.34	0.63	0.004	0.32	-

*LTZ* letrozole group, *CON* control group, *h* hours. ^A−C^ in the control group and ^a−d^ in the letrozole group without a common superscript denotes a significant difference (*P* < 0.05) within that group. *Refers to a significant difference (*P* < 0.05) between groups (LTZ vs. CON) at that time point

### Effect of LTZ on semen quality

The influence of LTZ administration on semen quality parameters at the experimental time points (0, 48, and 168 h) is presented in Table 2. Semen volume was affected by LTZ administration (*P* < 0.01), time (*P* < 0.01), and LTZ administration*time interaction (*P* < 0.01). For instance, semen volume increased (*P* < 0.01) at 48 h post-LTZ administration but diminished at 168 h (*P* > 0.05) to just before administration (0 h) in the LTZ group. Semen volume was higher at 48 (*P* < 0.05) post-LTZ administration compared to the CON group. On the other hand, there were no significant differences among both groups at 168 h. Mass motility was affected by time (*P* < 0.05), and LTZ* time interaction (*P* < 0.01). In the LTZ group, mass motility was higher (*P* < 0.01) at 48 h post-LTZ administration compared to 168 h. Mass motility score in the LTZ bucks was higher (*P* < 0.01) at 48 h post-administration than in the CON, while at 168 h CON group was higher (*P* < 0.05) than LTZ. For individual motility, there were effects of time (*P* < 0.01) and LTZ administration* time interaction (*P* < 0.05). In the LTZ group, individual motility increased (*P* < 0.05) at 48 h, then declined (*P* < 0.01) at 168 h post-administration. Individual motility percent was higher (*P* < 0.01) in the LTZ bucks at 48 h post-administration than in the CON, while no significant differences at 168 h among both groups. Sperm viability was affected by LTZ administration, there was a time (*P* < 0.01) effect. At 48 h post-LTZ administration, sperm viability percent was higher (*P* < 0.05) in the LTZ bucks than in the CON bucks, but at 168 h there was no significant difference. In addition, spermatozoa with normal morphology were affected by time (*P* < 0.01). In the LTZ bucks, normal sperm percent elevated (*P* < 0.05) at 48 and 168 post-administrations compared to 0 h. In comparison to CON, LTZ bucks had higher normal sperm percent at 48 h administration (*P* < 0.01), though at 168 h showed no significant differences between the two groups. Finally, semen pH values were not affected either by LTZ administration, time, or their interaction.

## Discussion

Our objective was to elucidate the acute effect of LTZ as an effective aromatase inhibitor on testicular blood flow and sperm quality in goat bucks under HS conditions. In this experiment, the LTZ group showed a rapid and transient increase in T concentrations as well as an improvement in sperm quality. In our study, there was a significant augmentation in T concentration at 2, 24, and 48 h in the treated group which was associated with E2 suppression from 2 h to 48 h. Hence, LH increased at two time points (2 and 72 h). It could be related to the LTZ effect as the peak plasma concentration of LTZ reached shortly following administration as reported in anestrous ewes and humans (Jin et al. 2012; Kivrak et al. 2021). The finding that treatment with LTZ-induced elevation in T level and E2 suppression in the present study could be probably due to blockage of the conversion of T to E2 resulting in T accumulation (Verma and Krishna, 2017) or through the negative feedback effect on the hypothalamic-pituitary axis that triggered increasing in LH release, consequently enhancing T production (Oduwole et al. 2021; Odetayo et al. 2024). The temporary effect of LTZ on LH release at 2 and 72 h might be attributed to LTZ effect on LH rhythm or due to the negative feedback effect of T. In this aspect, Kivrak et al. (2021) and Colleluori et al. (2020) documented a deficiency in the alteration of LH concentration following aromatase inhibitors administration. Furthermore, an in vitro study (Verma and Krishna 2017) proposed that increasing in T level might not associated with the expression of steroidogenic enzyme or LH stimulation. In boars, dealing with aromatase inhibitors showed no change in gonadotrophin secretion, despite E2 reduction (At-Taras et al. 2006). Therefore, further investigation is needed to clarify the LTZ action on LH rhythm.

Semen volume, sperm mass motility, individual motility, and sperm viability increased in the LTZ group at 48 h post-LTZ administration compared to the CON group. Furthermore, the ejaculation time and total sperm abnormalities were reduced in the LTZ group compared to the CON. Our results are consistent with previous studies in goat bucks (Rezaei et al. 2022), men (Cavallini et al. 2011; Schlegel 2012; Peivandi et al. 2019; Kooshesh et al. 2020), and male rats (Turner et al. 2000). Although, in goat bucklings semen volume, sperm concentration, total sperm per ejaculate, and semen index showed marked improvement, while semen pH, sperm progressive motility, viability, abnormality, acrosome integrity, and membrane integrity were unchanged (Rezaei et al. 2020). We supposed that the improvement in semen parameters post-LTZ administration might be related to the direct suppression of E2 by LTZ, which consequently increased the production of T( Kooksh et al. 2020; Naelitz et al. 2023) resulting in improving the quality of spermatozoa stored in the epididymis and altered seminal plasma composition (Davis and Pearl 2019; Menad et al. 2021 ) by enhancing the production and release of proteins, fructose, citric acid, some ions, and enzymes by the accessory sex glands into the seminal plasma (Druart X and de Graaf 2018; Recuero et al. 2019). Altogether, all these elements in seminal plasma interact with the sperm membrane to exert a favorable impact on sperm metabolism, motility, capacitation, and fertilization potential (Rodriguez-Martinez et al. 2021). Hence, this noticed response after a short time of LTZ administration was probably not linked to spermatogenesis as it required 49 days for full spermatogenesis to happen. Thus, these enhancements were more likely related to the enhancement in the conditions of sperm stored in the epididymis and seminal plasma constituents. On the contrary, LTZ had negative impacts on the semen parameters of Capra hircus goats (Ortiz-Carrera et al. 2019), which could be attributed to different breeds or doses and routes of LTZ administration, so further investigation is needed.

Regarding our finding for the increase in sperm motility post-LTZ administration, this could be attributed to T elevation inducing glucose, lactate, glucose transporter (GLUT8), and steroidogenic acute regulatory protein (StAR) expression (Banerjee et al. 2014). Besides, fructose and glucose increased sperm ATP concentration, motility, and sperm plasma membrane integrity as reported in roosters (Getachew et al. 2015) and mammals (Amaral 2021). Moreover, T is considered an aromatization substrate causing increasing in sperm movement, promoting energy, and acrosome reaction (Guo et al. 2021). However, the mRNA level of P450arom was lesser in non-motile spermatozoa than in motile spermatozoa, considering that aromatase might be associated with motility (Lambard et al. 2003).

On the other hand, the reduction in abnormal morphology by LTZ is incongruent with Kooshesh et al. (2020) who found an appropriate sperm formation following LTZ administration, that could be relayed to the improvement of DNA fragmentation due to the suitable altering of histone with protamine, besides the anti-apoptotic pathway under the control of sex hormones (Sakkas et al. 2018). In this study, high ejaculate volumes of LTZ-treated bucks might be associated with the increase of the accessory gland’s activity and epididymis in response to elevated T release. The accessory glands are thought to be androgen-dependent structures and most of the seminal plasma is made up of their secretions. It’s well documented that aromatase was found in the epididymis, seminal, and prostate glands of goats (Farzinpour et al. 2015). Thus, the inhibition of aromatase might increase local androgen that is not converted to E2 perhaps enhancing the secretions of the reproductive tract into the seminal fluid including biochemical components and other vesicular products (Getachew et al. 2015).

Continually, the reduction of ejaculation time in LTZ-treated male goats could be linked to the elevation in T. As reported higher serum T levels positively impacted the ejaculation time in male goats (Ángel-García et al. 2015). Likewise, T binds to androgen receptors which act on the central nervous system to prompt sexual behavior by increasing the frequency of courtship, mount, ejaculation, and self-urination (Fritz et al. 2019). Therefore, the use of LTZ could have a promising effect on overcoming the concerns of animal welfare about the negative impact of using electro-ejaculator as LTZ decreases the ejaculation time, which is accompanied by reducing the pain and the stress imposed on the animal during the time of semen collection. In the present study, at 168 h some semen parameters such as mass motility, individual motility, sperm viability, and normal morphology showed no significant difference among both groups. This could be elucidated by the administration of a single dose of LTZ that was characterized by stabilized plasma concentrations kept for certain times but lacked continuous accumulation (Arora et al. 2019). Besides, the short half-lifetime of LTZ (48 h) rapidly disappears from circulating blood (Jin et al. 2012; Mukherjee et al. 2021). In addition to the stressful effect of HS on bucks; thus, further studies are required to display the effect of LTZ in repeated doses and different conditions.

In this experiment, heart rates were reduced at 2 h and 96 h in treated bucks, which might indicate a possible effect of LTZ on heart rate. Goats exposed to HS showed an elevation in heart rate from 74 to 91 beats/min (Okoruwa 2014; Ribeiro et al. 2018). Contrarily, heart rate revealed no changes during HS (Sunagawa et al. 2002). Intriguingly, it decreased from 115.7 to 85.8 beats/min as reported by Al-Haidary (2004) in sheep. Rectal temperature and respiratory rate are indices reflecting thermal regulation and HS conditions that adversely affect growth performance and reproduction (Pragna et al. 2018; de La Salles et al. 2020). Goats could keep their rectal temperatures below 38.5 °C at rest (El-Tarabany et al. 2017). Our results agree with (Okoruwa 2014; Wang et al. 2016) who reported an increase in temperature from 37 ℃ to 41 ℃ during HS. Phulia et al. (2010) and Shilja et al. (2016) also reported a temperature boost from 38.8 to 39.5 °C during high temperatures. As previously reported respiration above 12 to 20 breaths /minute denoted HS (Okoruwa and Ikhimioya 2014). Possibly a single dose of LTZ couldn’t affect respiratory and rectal temperature in a short time; therefore, multiple doses are required to ensure LTZ impact.

Concerning hemodynamics, LTZ exhibited no change in blood flow throughout the study except at 96 h Doppler indices were reduced in the LTZ group compared to CON. Previously, LTZ did not affect uterine artery hemodynamics in baboons, women, and pregnant equine respectively (Aberdeen et al. 2010; Sakhavar et al. 2014; Esteller-Vico et al. 2017). Furthermore, in previous studies, LH and FSH injections couldn’t alter total blood flow to the testis (Lindner 1961; Amann et al. 1978). Also, injection of human chorionic gonadotropin into anesthetized rams increased T secretion but did not affect testicular blood flow (Lindner 1969; Damber et al. 1992). Moreover, no significant changes were reported post-FSH administration in testicular blood flow throughout different time points, nevertheless RI and PI decreased at 48 h (Samir et al. 2023). This explanation might be related to the indirect effect of LTZ on the T level causing a reduction in RI, PI, and S/D at 96 h. In contrast, direct injection of T in rats normalized testicular blood flow and vasomotion (Kivrak et al. 2021), we assumed that unchanged blood flow might reflect the indirect action of LTZ on testicular blood vessels or related to the only given dose of LTZ to bucks under HS conditions. So, further research is needed to elucidate the role of LTZ upon testicular blood flow in long-term treatment, different doses, other species, and different conditions.

This study had some limitations. We only had 12 bucks; they were all of the same breed. Furthermore, we did not measure the LTZ level during the experimental period. Despite those limitations, there were significant differences between CON and treated groups which were generally consistent with previous reports utilizing LTZ. Therefore, although this study should be replicated (with care to overcome the stated limitations), the results were clear and arguably compelling.

## Conclusion

Administration of single dose LTZ (S/C) improved semen quality within 48 h and reduced the ejaculation time as well as enhanced T levels in goat bucks during HS conditions. However, further studies are required to elucidate the LTZ mechanistic action on semen quality in large goat populations in different environmental conditions.

## Data Availability

No datasets were generated or analysed during the current study.
